# Clinical Comparison of FD-CT and MS-CT in Aneurysmal Subarachnoid Haemorrhage: A Single Center Experience

**DOI:** 10.3390/diagnostics12102443

**Published:** 2022-10-09

**Authors:** Felix Eisenhut, Cornelius Heidelbach, Elisabeth Heynold, Michael Manhart, Tobias Struffert, Sebastian Brandner, Arnd Doerfler, Stefan Lang

**Affiliations:** 1 Department of Neuroradiology, University Hospital Erlangen, Friedrich-Alexander University Erlangen-Nuremberg, Schwabachanlage 6, 91054 Erlangen, Germany; 2 Department of Neurosurgery, University Hospital Erlangen, Friedrich-Alexander University Erlangen-Nuremberg, Schwabachanlage 6, 91054 Erlangen, Germany; 3 Advanced Therapies, Innovation, Siemens Healthcare GmbH, Siemensstraße 1, 91301 Forchheim, Germany; 4 Department of Neuroradiology, University Hospital Giessen, Klinikstraße 33, 35392 Gießen, Germany

**Keywords:** cerebral aneurysm, endovascular treatment, flat-detector computed tomography, multislice computed tomography, subarachnoid haemorrhage

## Abstract

Single-center comparison of postinterventional multislice computed tomography (MS-CT) and flat-detector computed tomography (FD-CT) in patients with subarachnoid haemorrhage (SAH) and endovascularly treated cerebral aneurysms with a focus on detection of posttherapeutical complications. Patients with endovascularly treated aneurysmal SAH undergoing both MS-CT and FD-CT within 24 h after intervention were included. Datasets were compared regarding image quality (IQ) as well as qualitative (detection of SAH, intracerebral haemorrhage [ICH], intraventricular haemorrhage [IVH], external ventricular drain [EVD] position, acute obstructive hydrocephalus [AOH]) and quantitative (cella media distance [CMD], modified Graeb score [GS]) parameters. 410 patients with endovascularly treated aneurysmal SAH were included. IQ was equal between MS-CT and FD-CT. FD-CT allowed equal detection of SAH and ICH in comparison to MS-CT. FD-CT allowed excellent detection of IVH and delineation of EVD position with strong agreement to MS-CT findings. FD-CT allowed equal detection of AOH in comparison to MS-CT. There was no significant difference of CMD and GS between FD-CT and MS-CT. Postinterventional FD-CT yields equivalent diagnostic value in patients with endovascular treated SAH as MS-CT. Enabling reliable detection of SAH-associated complications within the angiosuite, FD-CT might be an efficient and safe imaging modality in these clinical emergencies.

## 1. Introduction

Despite effective therapeutical options for aneurysm treatment (e.g., endovascular coiling), acute aneurysmal subarachnoid haemorrhage (SAH) remains a life-threatening condition associated with high morbidity and mortality [1,2,3,4] with a survival rate of only 65% [5]. Following the typical clinical symptoms (sudden thunderclap headache in combination with vomiting, confusion or meningism) [6,7], immediate non-contrast cerebral computed tomography (cCT) with its excellent sensitivity (0.987) and specificity (0.999) [8] is the standard imaging modality for fast and reliable detection of SAH [9]—possibly supplemented by a lumbar puncture, if initial cCT is unobtrusive.

Then, prompt treatment of the ruptured aneurysm causing the SAH is indicated [10]: Both endovascular treatment using coils, stents, intra/extraaneurysmal flow-diversion or combinations of those and surgical clipping are viable treatment options depending on aneurysm location, size and configuration [11,12,13,14]. However, further close monitoring of the patients, who are typically under 60 years and female [15], is essential to identify SAH-associated complications [16] such as re-bleeding of the ruptured aneurysm with the highest risk in the first few hours after the initial event or acute obstructive hydrocephalus (AOH) with the highest risk within 48 h or prolongated after weeks or even a month in 20–30% of patients with SAH [17,18,19] and with consecutive need for an external ventricular drain [EVD]. Further complications are cerebral vasospasm with the highest risk between day 3 to 21 after SAH [20] and consecutive cerebral ischemia [21,22].

Therefore, prompt imaging control in patients with treated aneurysmal SAH is indicated. In this context, either standard postinterventional multislice computed tomography (MS-CT) with the necessity of patient transportation from the angiosuite to the scanner and consecutive transportation-associated efforts and risks (e.g., dislocation of the endotracheal tube or the EVD) or flat-detector computed tomography (FD-CT) directly within the angiosuite can be performed. Initial studies comparing MS-CT and FD-CT in the clinical routine demonstrated promising results regarding the diagnostic power of FD-CT for visualization of SAH, intracerebral haemorrhage (ICH), intraventricular haemorrhage (IVH) as well as the exact position of the EVD [23,24].

Motivated by these findings, we want to assess the diagnostic value of FD-CT in a large cohort of patients with SAH regarding detection of SAH, ICH, IVH as well as determination of EVD position and ventricle size using the cella media distance (CMD) in comparison to standard MS-CT.

## 2. Materials and Methods

### 2.1. Patients

Patients with SAH (Hunt & Hess grade 1–5) and following endovascular treatment (coiling, stenting and coiling, intra/extraaneurysmal flow-diversion) from October 2008 to April 2020 were considered for our analysis. Only patients undergoing both FD-CT and MS-CT within 24 h after intervention were included. The mean time difference between postinterventional acquisition of FD-CT and MS-CT was 13.1 ± 8.8 h.

This study was performed according to the Declaration of Helsinki and the European Guidelines for Good Clinical Practice. Additional ethical review was not required for participation in this retrospective analysis in accordance with local legislation (BayKrG sect. 27 para. 4) and institutional requirements.

### 2.2. Acquisition and Postprocessing

#### 2.2.1. FD-CT

FD-CT was performed on a biplane angiography system (Axiom Artis Zee biplane, Siemens Healthcare GmbH, Erlangen, Germany) using a standard protocol (20sDR-H, Siemens Healthcare GmbH, Erlangen, Germany) with the following parameters: scan time 20 s, 217° total rotation angle, 0.4°/frame, about 400 slices, matrix 512 × 512, 70 kV, 1.2 μGy/frame. The raw FD-CT data was postprocessed on a dedicated workstation (Leonardo, Siemens Healthcare GmbH, Erlangen, Germany) running commercially available software (InSpace 3D, Siemens Healthcare GmbH, Erlangen, Germany). Standard reconstruction of FD-CT datasets was performed using the following parameters: kernel type ‘HU’, image impression ‘smooth’, mode ‘NatFill’, slice thickness 0.3 mm.

#### 2.2.2. MS-CT

MS-CT was performed on a commercially available scanner (SOMATOM Definition AS+ 128, Siemens Healthcare GmbH, Erlangen, Germany) using a standard brain CT protocol. MS-CT data was postprocessed using the H31s brain kernel.

Then, axial multiplanar reformations (MPR) of both FD-CT and MS-CT were compiled with a slice thickness and slice distance of 4 mm.

**Figure 1:**
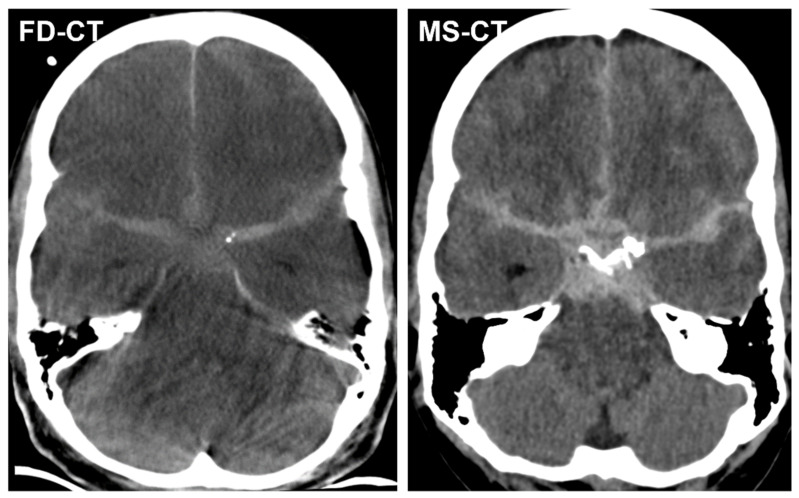
Exemplary flat-detector computed tomography (FD-CT) and multislice computed tomography (MS-CT) images of a patient with an endovascular treated, aneurysmal subarachnoid haemorrhage (SAH). Both FD-CT and MS-CT allow the delineation of the SAH as hyperdense filling of the subarachnoid spaces around the circle of Willis.

Figure 1 shows exemplary FD-CT and MS-CT images of a patient with endovascular treated SAH.

### 2.3. Data Evaluation

All datasets were analysed with commercially available viewing software (syngo.plaza VB30A, Siemens Healthcare GmbH, Erlangen, Germany) in consensus reading by two experienced neuroradiologists.

#### 2.3.1. Image Quality

Image quality (IQ) of all FD-CT and MS-CT datasets was evaluated by using a 5-fold scaled Likert scale: (0) not assessable due to artifacts; (1) severe artifacts, very limited assessability; (2) moderate artifacts, limited assessability; (3) minimal artifacts, good assessability; (4) no artifacts, excellent assessability.

#### 2.3.2. Qualitative Analysis

All FD-CT and MS-CT datasets were evaluated regarding the presence of a SAH, ICH and IVH (yes; no) as well as a correct intraventricular EVD position (yes; no) and the presence of an AOH (yes; no).

#### 2.3.3. Quantitative Analysis

CMD as the maximum external diameter of the bodies of the lateral ventricles (cm) was manually measured and compared in all FD-CT and MS-CT datasets.

The modified Graeb score (GS) as semiquantitative parameter for IVH volume measurement [25] was determined and compared in all FD-CT and MS-CT datasets.

### 2.4. Statistical Analysis

IQ, CMD and GS were analysed by use of descriptive statistics and tested for normal distribution by using the D’Agostino-Pearson test (if *p* > 0.05, normality was accepted). Then all values were tested for a significant difference between FD-CT and MS-CT datasets via an unpaired, two-tailed *t*-test (if values showed a Gaussian distribution) or via the Mann-Whitney-U (if values showed no Gaussian distribution).

Statistical analysis was performed with GraphPad Prism 9 (GraphPad Software, San Diego, CA, USA) and Excel (Microsoft, Redmond, WA, USA).

## 3. Results

### 3.1. Patients

In total, 410 patients (n_female_ = 271 [66.1%], n_male_ = 139 [33.9%], age_mean_ = 54.3 ± 13.1 years) with SAH and following endovascular treatment of a ruptured intracranial aneurysm were included in our retrospective analysis. In 388 patients a singular aneurysm was treated, in 21 patients 2 aneurysms and in one patient 3 aneurysms were treated.

Table 1 summarizes patient characteristics including Hunt & Hess grade [26], the endovascular treatment approach as well as aneurysm characteristics.

### 3.2. Image Quality

#### 3.2.1. FD-CT

In 326 FD-CT datasets IQ was rated as ‘4’, in 56 datasets as ‘3’, in 20 datasets as ‘2’ and in 8 datasets as ‘1’. No FD-CT dataset was rated as ‘0’. The mean IQ of FD-CT of all evaluated datasets was 3.71 ± 0.65.

#### 3.2.2. MS-CT

In 330 MS-CT datasets IQ was rated as ‘4’, in 52 datasets as ‘3’, in 21 datasets as ‘2’ and in 7 datasets as ‘1’. No MS-CT dataset was rated as ‘0’. The mean IQ of MS-CT of all evaluated datasets was 3.72 ± 0.64.

There was no significant difference between IQ of FD-CT and MS-CT (IQ_FD-CT mean_ = 3.71 ± 0.65, IQ_MS-CT mean_ = 3.72 ± 0.64, *p* = 0.7422).

**Figure 2:**
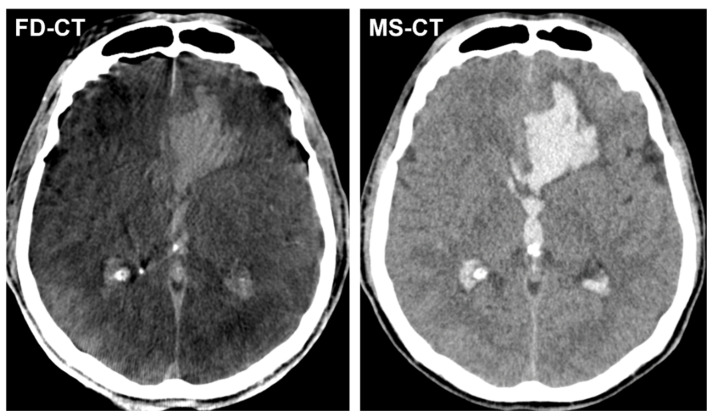
Exemplary FD-CT and MS-CT images of a patient with an endovascular treated, aneurysmal SAH and accompanying atypical intracerebral haemorrhage (ICH) in the left frontal lobe as well as intraventricular haemorrhage (IVH) in both lateral ventricles and the third ventricle. Both FD-CT and MS-CT allow equal delineation of ICH and IVH.

Figure 2 shows exemplary FD-CT and MS-CT images of a patient with ICH and IVH.

**Figure 3:**
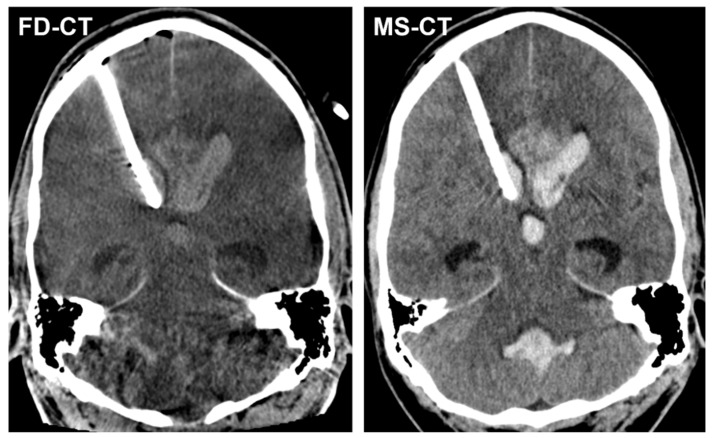
Exemplary FD-CT and MS-CT images of a patient with an endovascular treated, aneurysmal SAH and implanted external ventricular drain (EVD) because of acute obstructive hydrocephalus (AOH). Both FD-CT and MS-CT allow the precise determination of the intraventricular EVD position in the frontal horn of the right lateral ventricle as well as the delineation of the dilated ventricular system.

Figure 3 shows exemplary FD-CT and MS-CT images of a patient with an implanted EVD because of AOH following aneurysmal SAH.

Table 2 summarizes IQ evaluation of FD-CT and MS-CT.

### 3.3. Qualitative Analysis

#### 3.3.1. Blood Distribution

There was no difference between FD-CT and MS-CT regarding SAH detection: in 401 patients both FD-CT and MS-CT showed a SAH; in 9 patients lumbar puncture was used to detect SAH.

There was no difference between FD-CT and MS-CT regarding ICH detection: in 94 patients both FD-CT and MS-CT showed an ICH.

There was a difference between FD-CT and MS-CT regarding IVH detection: FD-CT showed an IVH in 309 patients; MS-CT showed an IVH in 281 patients.

#### 3.3.2. External Ventricular Drain Position

In total 302 of 410 patients had an EVD inserted. FD-CT showed a correct intraventricular EVD position in 299 patients; MS-CT showed a correct intraventricular EVD position in 290 patients.

#### 3.3.3. Acute Obstructive Hydrocephalus

There was no difference between FD-CT and MS-CT regarding AOH detection: Both FD-CT and MS-CT showed an AOH in 290 patients.

Table 3 summarizes the qualitative analysis regarding blood distribution, EVD position and detection of AOH.

### 3.4. Quantitative Analysis

#### 3.4.1. Cella Media Distance

There was no significant difference between CMD in FD-CT and MS-CT (CMD_FD-CT_ = 2.32 ± 0.63 cm, CMD_MS-CT_ = 2.24 ± 0.65; p_CMD_ = 0.0694).

**Figure 4:**
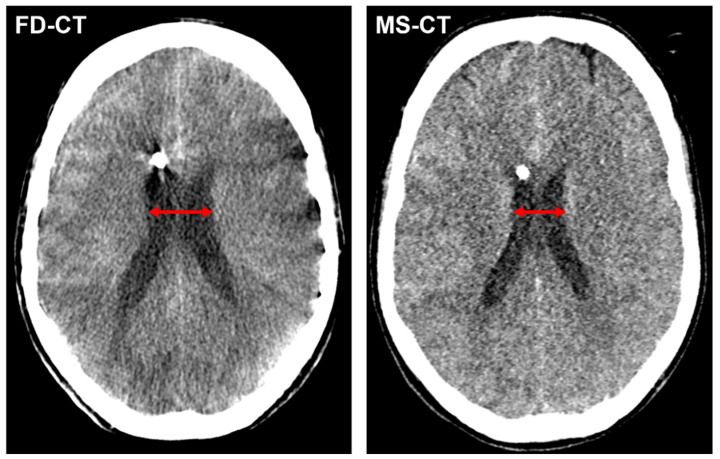
Exemplary cella media distance (CMD) measurement (red arrow) in a FD-CT and MS-CT dataset of a patient with endovascular treated, aneurysmal SAH and slightly declining width of the lateral ventricles from postinterventional FD-CT to MS-CT.

Figure 4 shows an exemplary CMD measurement in a FD-CT and MS-CT dataset.

#### 3.4.2. Modified Graeb Score

There was no significant difference between GS in FD-CT and MS-CT (GS_FD-CT_ = 3.22 ± 3.05, GS_MS-CT_ = 2.90 ± 2.99, p_GS_ = 0.0857).

## 4. Discussion

Aneurysmal SAH and its associated complications still remain a life-threatening condition and neurologic emergency [9]. Following the initial event, patients with aneurysmal SAH usually require continuous monitoring and timely aneurysm treatment. Furthermore, in addition to clinical monitoring radiologic follow-up is essential to ensure fast detection of clinical worsening due to rebleeding, cerebral vasospasm or AOH [2], especially in intubated and clinically not assessable patients.

In this context, we wanted to compare the diagnostic value of postinterventional FD-CT and standard MS-CT as follow-up examination in a large cohort of patients with endovascular treated aneurysmal SAH in the first 24 h after the initial bleeding. Demonstrating equal IQ, both imaging modalities equally allowed the reliable assessment of intracerebral blood distribution and EVD position as well as AOH detection. Reducing the need for patient transportation, saving time and manpower, FD-CT—available immediately postinterventional within the angiosuite—turned out as a reliable and safe postinterventional imaging approach in these clinical emergencies.

These results are in accordance with previous findings: In a small series of 44 patients with ICH, Struffert et al. tested the reliability of FD-CT in the angiosuite in comparison to MS-CT. They found FD-CT nearly as reliable as MS-CT for ICH detection [23]. In a consecutive study in 2010, the authors also evaluated the power of FD-CT for visualization of SAH, IVH and the EVD in 65 patients. On the one hand, they again reported reliable delineation of ICH and the EVD. On the other hand however, FD-CT also showed limitations especially with regard to the detection of perimesencephalic SAH and diminutive IVH in the occipital horns [24]. We cannot reproduce these findings in our study as FD-CT was equal to MS-CT regarding the detection of SAH. Furthermore, FD-CT even allowed the more frequent visualization of IVH in 309 patients in comparison to MS-CT with IVH in 281 patients—probably due to the regularly delayed MS-CT imaging in the clinical routine. The different results regarding the diagnostic value of FD-CT between Struffert et al.’s analysis and our work might be attributable to the technical progress of FD-CT since 2010 with improvement of soft tissue contrast and reduction of artifact susceptibility: In this context, Eckert et al. evaluated FD-CT with a newly implemented reconstruction algorithm in 2017 regarding intracerebral blood detection. In their study, this optimized FD-CT turned out as a reliable tool for delineation of supratentorial ICH and IVH, but demonstrated limited value in infratentorial ICH and perimesencephalic SAH [27]. In contrast, FD-CT allowed equal visualization of supra- and infratentorial ICH as well as perimesencephalic SAH compared to MS-CT in our analysis. This is also in accordance with a study of Leyhe et al. reporting a sensitivity of 95%, 97% and 100% and a specificity of 97%, 100% and 99% for SAH, IVH and ICH detection via FD-CT in the angiosuite, respectively [28].

Our results regarding the excellent EVD delineation provided by FD-CT are also supported by previous studies: For example, Psychogios et al. described FD-CT as a reliable tool for the confirmation of correct catheter placement with no difference to MS-CT [29]. In accordance to these findings, Struffert et al. also reported good visibility of the EVD in FD-CT only limited in 16.7% of their patients due to motion artifacts and insufficient visibility of the ventricle margins [24]. The discrepancy between FD-CT and MS-CT regarding the EVD position in our study (299 patients with correct EVD position in FD-CT; 290 patients with correct EVD position in MS-CT) might be due to a secondary catheter dislocation—a poorly described, yet not uncommon complication in busy intensive care units [30]. Furthermore, FD-CT also enabled the safe detection of an AOH equal to MS-CT in 290 of our 410 included patients with a SAH. In this context, the small, not significant difference of the CMD between FD-CT and MS-CT in our series (CMD_FD-CT_ = 2.32 ± 0.63 cm, CMD_MS-CT_ = 2.24 ± 0.65; p_CMD_ = 0.0694) might be accountable to the different scanning time points of both imaging modalities: Whereas FD-CT is performed immediately postinterventional within the angiosuite, the MS-CT scan is usually regularly delayed several hours (on average, MS-CT was performed 13.1 h after the FD-CT in our study)—enough time to reduce the CMD in patients with an AOH and implanted EVD.

This study has some limitation: First, the retrospective evaluation of the included datasets. In this context, the two independent readers were aware of the exclusive inclusion of patients with a SAH. Thus, the validity of the finding of equal detection of SAH in FD-CT and MS-CT might be limited. However, the presence of coils, stents or EVDs in all FD-CT and MS-CT datasets does probably not allow complete blinding of both readers regarding the pathology to be found. Second, the single-center approach of our analysis. Third, the time difference of up to several hours between FD-CT and MS-CT scanning (mean time difference 13.1 h). Fourth, the manual measurement of CMD with the associated risk of minimal measurement errors.

## 5. Conclusions

Postinterventional FD-CT is equal to MS-CT regarding the delineation of SAH, ICH and IVH, the determination of the EVD position and detection of AOH in patients with endovascular treated SAH. Hence, FD-CT available within the angiosuite can improve the clinical workflow, saves time and manpower and might represent the more efficient and safe imaging approach in these clinical emergencies.

## Figures and Tables

**Table 1:** Patient and aneurysm characteristics.

Patients	n
male	139 (33.9%)
female	271 (66.1%)
**Hunt & Hess grade**	
1	127 (31%)
2	86 (21%)
3	71 (17.3%)
4	62 (15.1%)
5	64 (15.6%)
**Endovascular treatment approach**	
coiling	326 (75.3%)
ballon-assisted coiling	52 (12%)
extra-aneurysmal flow-diversion	25 (5.8%)
stent-assisted coiling	14 (3.2%)
intra-aneurysmal flow-diversion	12 (2.8%)
extra-aneurysmal flow-diversion plus coiling	4 (0.9%)
**Aneurysm location**	
anterior communicating artery	157 (36.3%)
internal carotid artery	110 (25.4%)
middle cerebral artery	45 (10.4%)
basilar artery	42 (9.7%)
posterior inferior cerebellar artery	27 (6.2%)
anterior cerebral artery	24 (5.5.%)
vertebral artery	15 (3.5%)
posterior cerebral artery	11 (2.5%)
anterior inferior cerebellar artery	2 (0.5%)

**Table 2:** Evaluation of image quality (IQ).

IQ	0	1	2	3	4
**n_FD-CT_**	0	8	20	56	326
**n_MS-CT_**	0	7	21	52	330

**Table 3:** Qualitative evaluation of blood distribution, EVD position and AOH in postinterventional FD-CT and MS-CT.

	n_FD-CT_	n_MS-CT_
**SAH**	401	401
**ICH**	94	94
**IVH**	309	281
**EVD_correct_**	299	290
**AOH**	290	290

## Data Availability

The data presented in this study are available on request from the corresponding author.
